# A Light Vehicle License-Plate-Recognition System Based on Hybrid Edge–Cloud Computing

**DOI:** 10.3390/s23218913

**Published:** 2023-11-02

**Authors:** Jiancai Leng, Xinyi Chen, Jinzhao Zhao, Chongfeng Wang, Jianqun Zhu, Yihao Yan, Jiaqi Zhao, Weiyou Shi, Zhaoxin Zhu, Xiuquan Jiang, Yitai Lou, Chao Feng, Qingbo Yang, Fangzhou Xu

**Affiliations:** 1 International School of Optoelectronic Engineering, Qilu University of Technology (Shandong Academy of Sciences), 3501 Daxue Road, Changqing District, Jinan 250300, China; jiancaileng@qlu.edu.cn (J.L.); cxy3796@163.com (X.C.); z15105429165@163.com (J.Z.); w806237081_666@163.com (C.W.); zhujianqun0127@163.com (J.Z.); yanyy202108@163.com (Y.Y.); 18668909463@163.com (J.Z.); 1205187303swy@gmail.com (W.S.); zhuzhaoxin3709@163.com (Z.Z.); jiangxiuquan6711@gmail.com (X.J.); 13062000810@163.com (Y.L.); cfeng@qlu.edu.cn (C.F.); 2 School of Mathematics and Statistics, Qilu University of Technology (Shandong Academy of Sciences), 3501 Daxue Road, Changqing District, Jinan 250300, China

**Keywords:** license plate recognition, model compression, edge computing, cloud computing

## Abstract

With the world moving towards low-carbon and environmentally friendly development, the rapid growth of new-energy vehicles is evident. The utilization of deep-learning-based license-plate-recognition (LPR) algorithms has become widespread. However, existing LPR systems have difficulty achieving timely, effective, and energy-saving recognition due to their inherent limitations such as high latency and energy consumption. An innovative Edge–LPR system that leverages edge computing and lightweight network models is proposed in this paper. With the help of this technology, the excessive reliance on the computational capacity and the uneven implementation of resources of cloud computing can be successfully mitigated. The system is specifically a simple LPR. Channel pruning was used to reconstruct the backbone layer, reduce the network model parameters, and effectively reduce the GPU resource consumption. By utilizing the computing resources of the Intel second-generation computing stick, the network models were deployed on edge gateways to detect license plates directly. The reliability and effectiveness of the Edge–LPR system were validated through the experimental analysis of the CCPD standard dataset and real-time monitoring dataset from charging stations. The experimental results from the CCPD common dataset demonstrated that the network’s total number of parameters was only 0.606 MB, with an impressive accuracy rate of 97%.

## 1. Introduction

With the rapid popularization and development of smart cities, real-time license plate detection is an important application in smart transportation. Today, the amount of data brought by large-scale IoT devices has surged, and this phenomenon has resulted in traditional centralized cloud server data processing facing problems such as high bandwidth, high latency, and low privacy. The accuracy and timeliness of license plate recognition (LPR) based on deep learning (DL) algorithms can meet the recognition tasks in many real-world scenarios. However, standalone versions of DL platforms have limitations, in particular with the increase of the data volume and data dimension [1]. To meet the computing demands of DL, a common approach is to utilize cloud computing. However, a large amount of data is uploaded to the cloud server, which can lead to risks such as network congestion or delay and brings more challenges to the user’s service quality and actual experience. Cloud computing models are widely used in intelligent monitoring systems to process various types of video and image data. However, they encounter bottlenecks in the actual LPR, mainly including (1) challenges in achieving ideal performance for vehicle and LPR real-time systems; (2) an elevated resource occupancy rate, with an inadequate availability of channel resources; (3) the transmission of video and image data possibly resulting in a significant increase in energy consumption. Edge computing (EC) presents a viable solution to address the nonuniform distribution of computational resources across individual edge nodes [2]. Intelligent monitoring systems employ edge-layer processing to achieve reduced latency and facilitate equipment miniaturization, which greatly reduce the required power consumption. In the future of 5G, a significant application scenario will be license plate identification systems based on EC. Edge computing allows data to be processed locally on edge servers close to the data source, and the network delay of data transmission is reduced, which is suitable for real-time data-processing scenarios. However, the current related research still has certain shortcomings in realizing real-time license plate recognition on edge devices. On the one hand, few studies have considered implementing real-time LPR applications on edge devices with limited computing power. On the other hand, complex vehicle recognition models are deployed on these resource-constrained edge devices. This situation can incur higher computational costs. When the task volume is large, large network delays will occur, resulting in tasks not being able to be processed in time.

Due to the rapid development of artificial intelligence (AI) technology, the computational power and complexity demanded by visual processing algorithms have significantly increased. This poses a serious challenge to current computer vision (CV) systems. The popular object-detection (OD) algorithms in DL include two-stage detection algorithms, which rely on anchor boxes, and one-stage detection algorithms, which also rely on anchor boxes [3]. The You Only Look Once (YOLO) series of OD algorithms is the most representative among these. The YOLO algorithm was proposed by Redmon [4], which divides images into grids and transforms the OD problem into a regression problem. For strengthened detection accuracy, as well as rapid detection network execution, the YOLOv7 neural network employs two-stage cascaded networks on machinery that performs superbly. The YOLOv7 method can achieve a balance between the recognition and determination of the speed and accuracy using graphics processing units (GPUs), such as those on typical traditional servers or PCs [5,6]. If algorithmic inference is performed on the edge gateway, the original network structure of YOLOv7 needs to be lightweight and compressed through advanced methods such as pruning, quantization, and distillation.

Complex license-plate-recognition systems generate large amounts of video images, and collecting and labeling all samples is difficult and time-consuming. The semisupervised learning (SSL) method is a popular machine learning paradigm and is an effective way to train large numbers of unlabeled and small numbers of labeled samples [7]. When the tag data are scarce, it can automatically use a small amount of ready-made tag data for pattern recognition, and the learning performance improves. The semiautomatic method of labeling license plate images was used in this paper, and a small amount of ready-made labeled data was used for pattern recognition to improve the learning performance [8].

The SSL-based EC model and lightweight LPR system are proposed in this paper, called Edge–LPR. The network structure of YOLOv7 is a redesigned and lightweight model [4], which is better suited for deployment on an edge gateway system. To address diverse real-time OD scenarios within the context of LPR systems, the training model was directly deployed on the edge gateway [9], and the LPR was directly performed on the edge gateway. In times of idleness, the data are automatically transferred to the cloud-based device. The device assumes the responsibility of filtering and categorizing freshly obtained data at the edge gateway and subsequently transmitting the data to revise the weights and model parameters [10].

The following are this paper’s key contributions:

(1) The semisupervised learning (SSL) method was used for semiautomatic license plate labeling. Manually labeling license plate data is a time-consuming and labor-intensive task. Therefore, the SSL method was introduced into the license-plate-recognition (LPR) process, and a small amount of labeled license plate data were used to directly generate labels for a large amount of unlabeled license plate data. Our method improved the efficiency of LPR.

(2) New and efficient Edge–LPR system: First, based on the YOLOv7 framework, an attention mechanism was introduced into the C3 module to enhance the perception ability and accuracy of the underlying network. At the same time, the head of YOLOv7 was improved, and the Level 3 detection was upgraded to Level 4 detection, which uses the fusion of features at each layer to identify targets of different sizes and improves the accuracy of small target detection. Finally, channel pruning was used to reconstruct the backbone layer, reduce the network model parameters, and effectively reduce the GPU resource consumption.

(3) A more efficient edge–cloud hybrid recognition system: Traditional cloud computing and hybrid edge cloud computing solutions were compared and evaluated. The training procedure of the proposed model can run on cloud workstations with strong computing capabilities. The training model can obtain new weight files. In the edge computing gateways, the update weight files are used to complete the edge recognition. The real-time computing and inference are performed on the edge gateway. The recognition speed of Edge–LPR can reach 187.6 FPS, and the recognition accuracy can reach 95.6%.

The remaining sections are structured in the following manner. The associated work of DL networks in LPR and in the creation of EC device implementation is introduced in Section 2. The Edge–LPR system’s architecture and algorithm are presented in Section 3. The experimental findings and a comprehensive description are presented in Section 4. The study’s results and potential applications are presented in Section 5.

## 2. Related Work

In this section, the recent advances in LPR technology are firstly discussed, and then an overview of the development of model compression technology is provided. Finally, the application of OD in EC is summarized.

### 2.1. License Plate Detection Algorithm

DL is used to create image features, and manually creating image features are the two main methods currently used by LPR systems for intelligent detection.

(1) A technique using fictitious visual features

By integrating edge statistics with mathematical morphology or seed growth methodologies, it is possible to ascertain the existence of the license plate from the image’s regular edges. The structural elements are visible because the license plate contains characters that make up a string. Therefore, by identifying characters in the photograph, the license plate can be located. Li. et al. [11] used the MSER method to extract character areas from a license plate. Li. et al. [12] proposed that pixel color is a key component of license plates. To identify actual license plates in candidate zones, a cascaded license plate classifier based on color salient features was developed. According to the identical distribution of pixel textures in the license plate area, texture features were used to determine the license plate location [13].

(2) Method based on DL technology

The LPR techniques that relied on visual features have run into development roadblocks because of a variety of issues, including complex settings and erratic image lighting. DL performs exceptionally well at detecting objects [14]. The multi-OD algorithm has significantly outperformed earlier systems. The LPR techniques based on DL had been suggested by Xie and Co. [15]. A multidirectional LPR framework built on CNN was proposed, called the MD-YOLO model. The distinction was that our approach concentrates on mobile applications, which can fully utilize computing resources and increase LPR efficiency.

### 2.2. License Plate Recognition

Two of the commonly used LPR phases are character categorization and character recognition. Character segmentation methods commonly used include projection algorithms, SIFT features, and extreme area extraction [16]. Because character directions in the image can be easily changed by blurring, noise, and distortion [17], the aforementioned methods suffer from segmentation bias, which causes false positives for recognition. Various other license plate character recognition techniques that do not require character segmentation were proposed by Gezdev et al. [18]. The lightweight LPR network LPRNet, which can produce recognition results rapidly and with exceptional precision, has been suggested for deployment.

### 2.3. Lightweight Object Detection Model

The usage of OD technology is widespread in fields of study such as rubbish sorting, autonomous driving, and vehicle identification. OD algorithms based on DL have gradually become mainstream. YOLO, single-shot multibox detector (SSD), and RetinaNet are examples of one-stage approaches. These methods employ the idea of regression, eliminate the preclassification and regression phase of the two-stage approaches, and directly separate specific categories while regressing the border. Two-stage approaches tend to be utilized to describe OD techniques based on region hypotheses; primarily, R-CNN series techniques are used such as Fast R-CNN, Faster R-CNN, Mask R-CNN. The LPR network on edge gateways necessitates not enhancing edge gateway memory and processing capabilities but optimizing regular neural networks. Model compression can be used to produce a reduced LPR model. M. Sandler [18] invented deep separable convolution, which dramatically reduces the number of characteristics in neural network modeling models to create a lightweight neural network model. Guan. et al. [19] developed a simple three-step detection framework module composed of a rough region scheme and a post-processing stage to identify obstacles in a single railway image. A simple approach to meter recognition that combines DL and conventional CV technology was proposed by Fan et al. [20]. On the basis of YOLOv4, Cai et al. [21] suggested a one-stage based OD system for autonomous driving. An optimization network pruning approach was put forth at the same time to address the issue of onboard computer resources. The computing platform was constrained and was unable to fulfill real-time demands.

### 2.4. Object Detection Application at the Edge Platform

**Figure 1:**
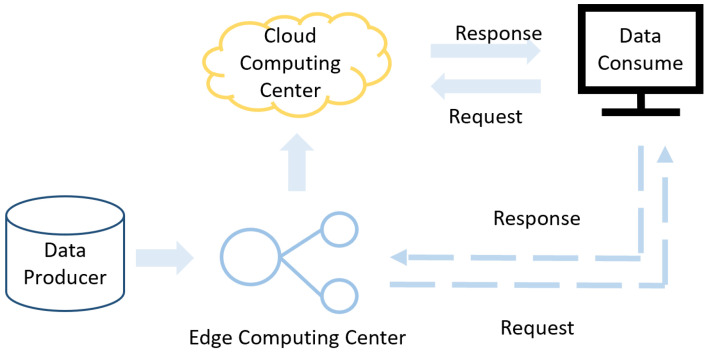
Comparison between edge computing and cloud computing.

EC evolved to deal with the negative aspects of cloud computing. Low latency, low bandwidth, and low cost were advantages of EC over cloud computing [22]. EC is closer to the data source, reducing latency, power consumption, and cost. The comparison between cloud computing and EC is shown in Figure 1. Wang et al. [22] reduced the model size from 64 MB to 8 MB through multiple sparse training and pruning techniques. Bi et al. [23] presented a YOLOv3-based dish identification machine on an FPGA architecture. To speed up the OD network and facilitate the deployment of edge platforms, Tu et al. [24] offered an improved slim instance segmentation system-based immediate-term defect detection scheme for tracking components.

SoC, FPGA, application-specific integrated circuits (ASIC), CPUs, and GPUs are examples of devices that could utilize EC. Initially, FPGA is considered to be the ideal hardware for EC adoption of AI. FPGA successfully scaled power consumption, but it is not great at inferencing performance or supporting the DL ecosystem. ASIC is suited for professional customization because of its lengthy development cycle and expensive cost [22], but it is challenging for it to adjust to the fast growth of object identification algorithms. The manufacturing of semiconductor components like GPUs, CPUs, and RAMs are proceeding apace; EC resources are provided to neural networks and increase the possibility of implementing AI models in EC. The NVIDIA Jetson series are promising AI SoC, which are more crucial to note [25]. They have high throughput, compact size, and good energy efficiency. Traditional GPUs need between 100 and 250 watts of power, but the integrated GPU in the Jetson uses between 5 and 15 watts. It can speed up model inference by converting popular DL frameworks like TensorFlow, Caffe, and Python into TensorRT [26] using transfer learning methods. The fundamental idea is to maximize the GPU’s capabilities, network layer fusion calculations, and inference accuracy [27]. It can offer high-throughput and low-latency deployment inference for embedded systems like safety monitoring and autonomous driving.

## 3. Method

This section provides a detailed introduction to the chosen system platform and design scheme. The YOLOv7 framework serves as the foundation, and an enhanced Edge–LPR algorithm is proposed in this paper, specifically tailored for edge gateways. The backbone layer was reconstructed by applying channel pruning to reduce the network model parameters. It effectively decreased the GPU resource consumption, while the feature fusion technology was enhanced to achieve a balance between high precision and real-time performance in the edge AI domain [28].

### 3.1. Comparative Design of System Platforms

**Figure 2:**
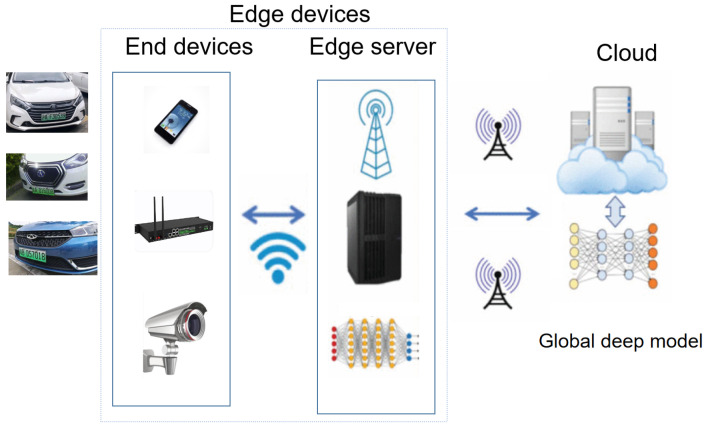
Schematic diagram of our edge cloud collaboration scheme.

OpenVINO was selected in this paper; it is a comprehensive tool suite launched by Intel, for the rapid deployment of applications and solutions. The model training task was placed on the cloud workstation with strong computing power. Cloud devices were trained regularly, and new weight files were obtained to update the model weights of edge gateways. At the same time, real-time Edge–LPR was performed on the edge gateway. Figure 2 shows the schematic diagram of our EC collaboration scheme.

(1) Real-time photographs of vehicles entering and exiting the charging post parking lot were captured by the camera (FPS was set to 30), and raw license plate information was allowed to be captured.

(2) The video stream was sent to the Intel Movidius Myriad X edge gateway device for license plate and model inference.

(3) The outcome was locally saved. The technology relayed the test findings at the same time.

(4) After labeling the data, the cloud server trained the data to obtain new weights and downloaded the new weights to the edge gateway.

### 3.2. License Plate Detection

In YOLOv7, FPN [29] uses upsampling and the fusion of various layer features to recognize targets at three different scales. The size of the vehicle in the photograph will be small if it is distant from the shooting location. The first 3-scale detection of the YOLOv7 head upgraded to 4-scale detection to solve this problem. To find targets, finer anchor boxes were used in larger feature maps. To determine the anchor box sizes for each target in the vehicle training set, the K-means clustering and intersection–union ratio were used by us (IoU, represented by $R_{I}oU$) with rectangular frames as a measure of similarity. Twelve sizes that matched four detection scales were picked [30]. The K-means clustering distance function is as follows:

(1) $$d\left(B,C\right)=1-R_{IoU}\left(B,C\right)$$

where *B* stands for the rectangle box’s dimensions, *C* for its center, and $R_{I}oU\left(B,C\right)$ for its intersection with another rectangular box.

**Figure 3:**
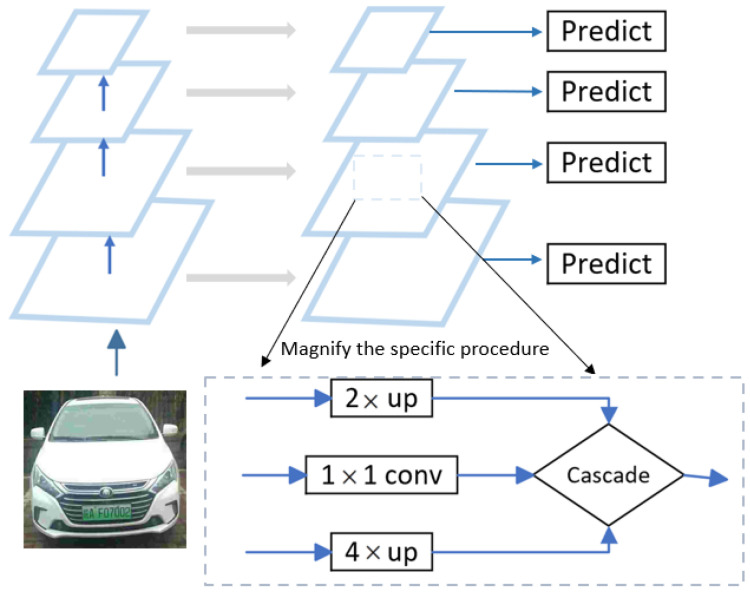
FPN architecture.

Figure 3 illustrates the use of a dense connection approach to fuse multiscale features while enhancing the original horizontal connection method for multiscale features. Cascade fusion, which preserved more of the original feature information, replaced parallel fusion. The semantic information of high-level functions and the detailed knowledge of low-level functions were utilized in this paper.

The image was divided by our detection network into an *S* grid. It made predictions for bounding boxes for each grid unit, and each box had a confidence rating. The likelihood of *C* conditional classes was predicted (the likelihood of object classes was one for each class). The network outputted the probability of class and offset value for each bounding box in turn. Features in the image were identified and bounding boxes with feature probabilities higher than a threshold were selected.

**Figure 4:**
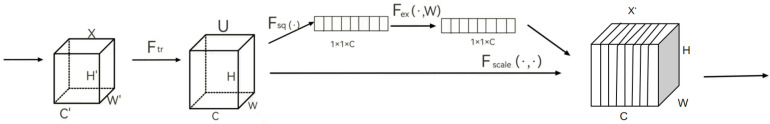
SENet network structure diagram.

The squeeze and excitation network (SENet) was used in the YOLOv7 C3 module to enhance the perception ability and accuracy of the underlying network [31]. The SENet schematic diagram is shown in Figure 4. The SENet evaluates the interaction between feature channels and applies an attention mechanism to them. First, the squeeze operation compresses the spatial dimension by globally pooling each feature map and obtaining an average value. This operation can include global receptive field features, which approximates a number and then enters it into the activation operation. Throughout the squeeze operation, the thread outputs a feature map of size 1 × 1 × C, and weights (w) are utilized to assess the exact correlation of C channels [32]. By reducing the dimensionality of the C channel, network computations can be reduced while improving nonlinearity capabilities. Additionally, SENet is an attention mechanism [33] that can improve the correlation between feature maps at various spatial places, and the model is enabled to pay closer attention to significant objects and regions.

**Figure 5:**
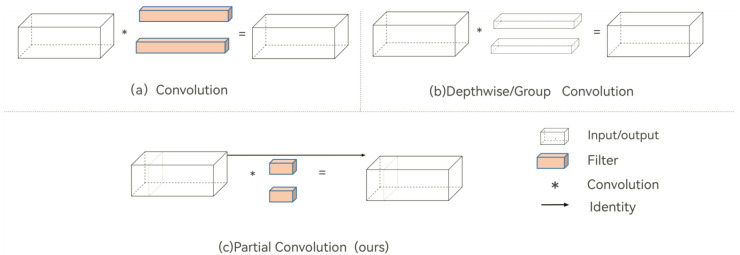
Comparison between PConv and conventional convolution, deep convolution/group convolution.

To enhance the perception ability and accuracy of using various levels of characteristics for prediction when LPR is a common practice, due to issues with license plate occlusion or large-degree tilt in the real-time monitoring of charging stations, the partial convolution (PConv) network was introduced to more accurately identify license plates. Figure 5 shows the comparison between PConv and traditional convolution, deep convolution/group convolution. Different levels of feature maps are treated by PConv as feature maps of different frames in the video frame [34], a method similar to 3D convolution. The output of the 3D convolution of the intermediate frames is obtained by performing 3D convolution on neighboring N frames. The information from the front and back feature layers are combined in the output, and the fusion process is more adaptable than simple addition or cascading. PConv performs quickly and effectively because it applies filters to a select few input channels without influencing the others. Compared to deep group convolution and ordinary convolution, PConv achieves smaller flops.

The target detector implemented as a sliding window introduces multiple detection anchors for the same target object. The NMS technology was employed to eliminate redundant bounding boxes in the license-plate-recognition process and to improve the accuracy of license plate recognition [35]. The NMS involves determining a threshold value ($T_{n}ms$) [29], a list of bounding boxes B is selected, and an appropriate confidence level *C* is set. The detection frame with the highest score is chosen initially. The IoU of the remaining detected frames is then compared to the IoU of the highest scoring frame. This process is repeated until the list *B* is empty. If there is a significant overlap between two target frames, the algorithm promptly removes the detection with the lower confidence level [36]. This approach may potentially lead to a low recall rate and missed detections; we utilized the Soft-NMS optimization process to adjust the score loss instead of immediately setting the score to 0 [37]. The final results were output using the IoU function. The NMS technology was used in this paper to eliminate redundant bounding boxes in the license-plate-recognition process and improve the accuracy of license plate recognition. Algorithm 1 describes the output process of the Soft-NMS algorithm.
**Algorithm 1** Soft-NMS method.
 **Input:** $B=\left\{b_{1},\cdots ,b_{N}\right\},C=\left\{c_{1},\cdots ,c_{N}\right\},T_{nms}$
*B* is a list of the initial detection boxes
*C* contains a list of corresponding detection scores
$T_{nms}$ relates to is the NMS threshold
 **Output:** Final detection Bounding box list *S*
1:function SOFT_NMS2:**while** B≠empty **do**3:   m←argmaxC4:   $S\leftarrow S\cup b_{m};B\leftarrow B\cup b_{m};C\leftarrow C\cup c_{m}$5:   $s_{i}\leftarrow s_{i}f\left(IoU\left(M,b_{i}\right)\right)$6:   **for** $b_{i}\in B$ **do**7:     **if** $IoU\left(M,b_{i}\right)\geq T_{nms}$ **then**8:        $B\leftarrow B\cup b_{m};C\leftarrow C\cup c_{m}$9:     **else**10:        $s_{i}\leftarrow s_{i}f\left(IoU\left(M,b_{i}\right)\right)$11:     **end if**12:   **end for**13:**end while**14:**return** result


### 3.3. Model Compression

**Figure 6:**
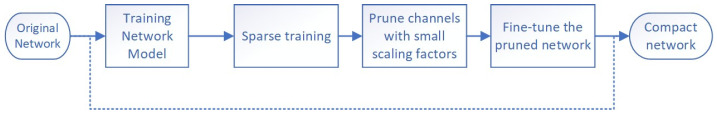
Example diagram of the pruning process.

YOLOv7s is a lightweight detection network, but the model is still relatively large, so it is necessary to reduce the network input size. But simply reducing the input to reduce the calculation, such as reducing from 640 to 320, will cause a great loss in the detection effect. The coefficients of the batch normalization (BN) layer can be constrained by adding L1 regularization to make the coefficients sparse. After sparse training, the layers with very small sparseness are cut out, and the corresponding activation is small, so the impact on the latter is very small [38]. By iterating this process repeatedly, a simplified model can be obtained. The entire pruning process is shown in Figure 6. First, the network was initialized, the parameters of the BN layer were regularized, and the network was trained. Then, the pruning rate was set to prune the network. By setting different pruning rates, redundant convolutional layers were removed and the model structure was optimized so that it can be better deployed on edge gateways. Finally, the pruned network was fine-tuned to complete the pruning work.

BN layer calculation:

(2) $$\hat{z}=\frac{z_{\tilde{in}}-\mu B}{\sqrt{\sigma^{2}+\varepsilon }}$$

(3) $$z_{\mathrm{out}\;}=\gamma \hat{z}+\beta$$

**Figure 7:**
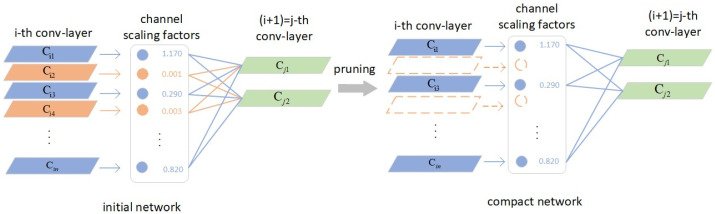
Schematic diagram of model pruning process.

The activation size of each channel is positively correlated with the coefficient (PyTorch corresponds to the weights of the BN layer, and β corresponds to the bias). If γ is closed to 0, the activation value is small. Sparse learning is performed based on the pretrained network, and the overall framework is shown in Figure 7. The design of lightweight networks is chosen for sparse learning to deliver a target number of parameters and computations[39]. Parameter values are initialized and retrained on the basis of the architecture created by the pruning process.

After training the network model of YOLOv7, the coefficients of the BN layers are similar to the normal distribution.

By adding L1 regular constraints:

(4) $$L=\sum_{\left(x,y\right)}l\left(f\left(x,W\right),y\right)+\lambda \sum_{\gamma \in \Gamma }g\left(\gamma \right)$$

where the loss function from regular training is the first item, and the second item is the constraint, where $g\left(s\right)=\left|s\right|$ , γ is the regularization coefficient. When performing backpropagation, parameters can be sparse and added to the training loss function:

(5) $$\begin{array}{c} L^{\prime }=\sum l^{\prime }+\lambda \sum g^{\prime }\left(\gamma \right)=\sum l^{\prime }+\lambda \sum {\left|\gamma \right|}^{\prime }\\ =\sum l^{\prime }+\lambda \sum \gamma *sign\left(\gamma \right) \end{array}$$

The first and second terms on the right side of the loss function are constraints, and *L* is the initial loss function. Where $g\left(s\right)=\left|s\right|$ , γ is the regular coefficient, it is necessary to multiply the weight of the BN layer by the output and coefficient of the coincidence function of the weight during backpropagation.

There are certain drawbacks to using complex convolutional networks in resource-constrained edge environments. It is difficult to leverage large networks for inference due to memory limitations, while lower computing power requires longer inference times [40]. The suggested pruning strategy can produce a network with the desired parameters and FLOPs, which makes it easier to employ CNN in embedded settings. Equations (6) and (7) represent the computation of the pruned network and parameter. $F_{l}$ , $P_{l}$, and $C_{l}$ represent the computation amount, number of parameters, and number of channels of the *l* convolutional layer, respectively. *L* is the number of layers in the network.

(6) $$F_{\mathrm{pruned}\;}=\sum_{l=1}^{L}\left\{F_{l}\left(\frac{\sum_{c=1}^{c_{l-1}}\theta \left(\gamma_{l-1,c},t\right)}{C_{l-1}}\right)\left(\frac{\sum_{c=1}^{C_{l}}\theta \left(\gamma_{l,c},t\right)}{C_{l}}\right)\right\}$$

(7) $$P_{\mathrm{pruned}\;}=\sum_{l=1}^{L}\left\{P_{l}\left(\frac{\sum_{c=1}^{c_{l-1}}\theta \left(\gamma_{l-1,c},t\right)}{C_{l-1}}\right)\left(\frac{\sum_{c=1}^{C_{l}}\theta \left(\gamma_{l,c},t\right)}{C_{l}}\right)\right\}$$

The same loss function as (8) is obtained by combining Equations (6) and (7). $F_{baseline}$ represents the calculation amount and the size of the baseline network’s parameters, and $F_{t\arg et}$ and $P_{t\arg et}$ represent the calculation amount and the number of parameters of the target pruning network, respectively. The indicator function developed is used by sparse learning, and $F_{{}_{pruned}}$ and $P_{pruned}$ signify the amount of computation and network parameters that are present after eliminating unneeded filters. The first bracket item on the right side of (8) and (10) indicates the trimmed effect of the previous layer, and the second bracket item indicates the trimmed effect of the current layer.

(8) $$Loss_{pruning}={\left(\frac{F_{pruned}-F_{t\arg et}}{F_{baseline}}\right)}^{2}+{\left(\frac{P_{pruned}-P_{t\arg et}}{P_{baseline}}\right)}^{2}$$

Cross-entropy loss is used for classification while training network parameters. The objective of this paper is to optimize the architectural parameters by gradient descent during training. Finally, the best-trimmed model is found, and the computational cost satisfies the resource limitations. The loss function has the following shape:

(9) $$\mathrm{Loss}=Loss_{origin}+\alpha Loss_{pruning}$$

for learning with the loss function of pruning $Loss_{pruning}$, and then they are solved simultaneously during training. The training is followed, and the ideal network design with the desired parameters and computation volume is established. Algorithm 2 describes the compression process of the Edge–LPR model.
**Algorithm 2** Compression of Edge–LPR algorithm.**Require:** output minimum loss *L*, maximum average accuracy AP**Ensure:** maximizing model accuracy1:initialization;2:input images set xi and its label *y*, training round *n*;3:train the previous network model T to obtain $\alpha_{T}$;4:conduct channel pruning and get reward AP, R;5:add L1 regular constraint,6:classify using cross entropy loss, $g\left(s\right)=\left|s\right|$;7:calculate the model accuracy Loss using Equations (8) and (10);8:calculate the model accuracy AP using Equations (15);9:**if** n<N **then**10:   repeat;11:   update the previous weight network model *T*;12:   end13:**end if**14:**if** n≥N **then**15:   update the previous weight network model *T*;16:   output *L*,AP;17:**end if**

### 3.4. License Plate Recognition

**Figure 8:**
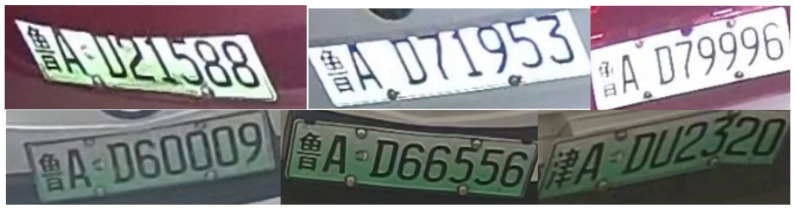
Camera-detected license plate image.

**Figure 9:**
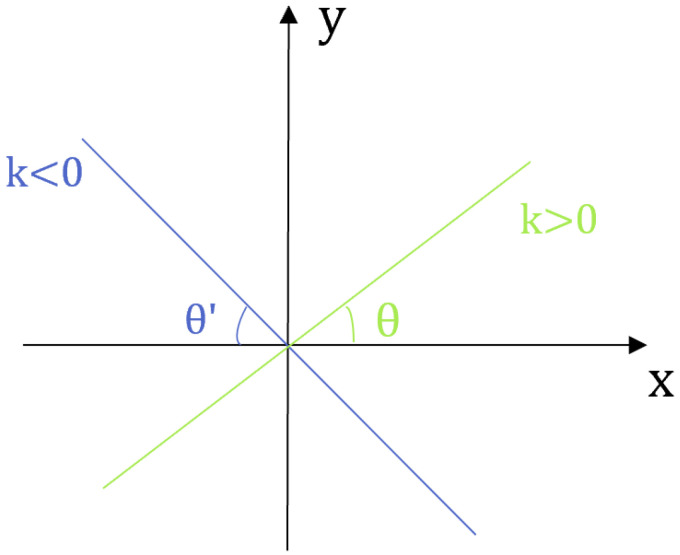
Schematic diagram of license plate rotation angle.

License plate images can be retrieved immediately through the Edge–LPR network, although these license plates are often tilted at an acute angle, as shown in Figure 8. The coordinate base was used as the center center to correct the horizontally rotated license plate image, as shown in Figure 9. Based on the license plate edge points received from the license plate positioning network, the slope of the longest edge in the license plate was used to calculate the tilt angle of the license plate. According to the positive and negative slope, it can be divided into two situations, as shown in the figure. When the slope is positive, the license plate must rotate clockwise. When the slope is negative, it should be rotated counterclockwise. If the slope of the longest side of the straight line is *k*, then the tilt angle of the license plate is θ. The inclination angle can be calculated using the following formula:

(10) θ=arctank

**Figure 10:**
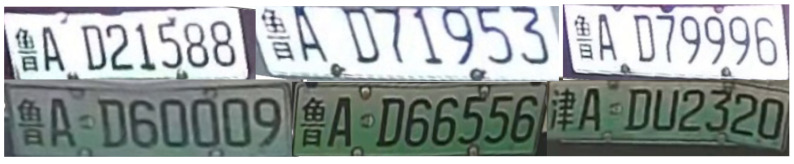
Corrected license plate image.

Given that the length of the line is *r*, and the initial angle is α, the line rotates about the origin of the point. The rotational angle is θ. Equations (11) and (12) can be used to determine the coordinates (m,n) following rotation while spinning clockwise. Figure 10 displays the results of the horizontal rotation correction for the license plate image. The illustration shows that, following correction, the license plate image with horizontal rotation can produce good results.

(11) m=rࢩcos(α−θ)

(12) n=rࢩsin(α−θ)

**Figure 11:**
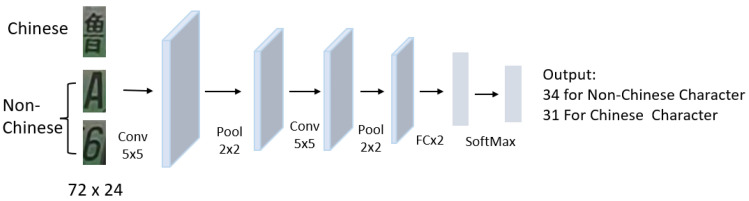
CNN-based LPR architecture.

End-to-end license plate character recognition is made possible by LPRNet, which uses a lightweight CNN structure with strong robustness and does not require license plate character segmentation [41]. Figure 11 shows the CNN-based LPR architecture. Three convolutional layers, three maximum aggregation layers, three basic modules, and two exit layers, used to avoid overfitting, make up the LPRNet backbone network. This network utilizes a convolutional layer as the output layer and a 94 × 24 picture as the input layer. The figure depicts its structure. An input layer, a feature output layer, and four convolutional layers make up each basic module. LPRNet uses a backbone network to extract image features in order to obtain the sequence of license plate characters and then a convolutional kernel for convolution.

### 3.5. Edge Computing

**Figure 12:**
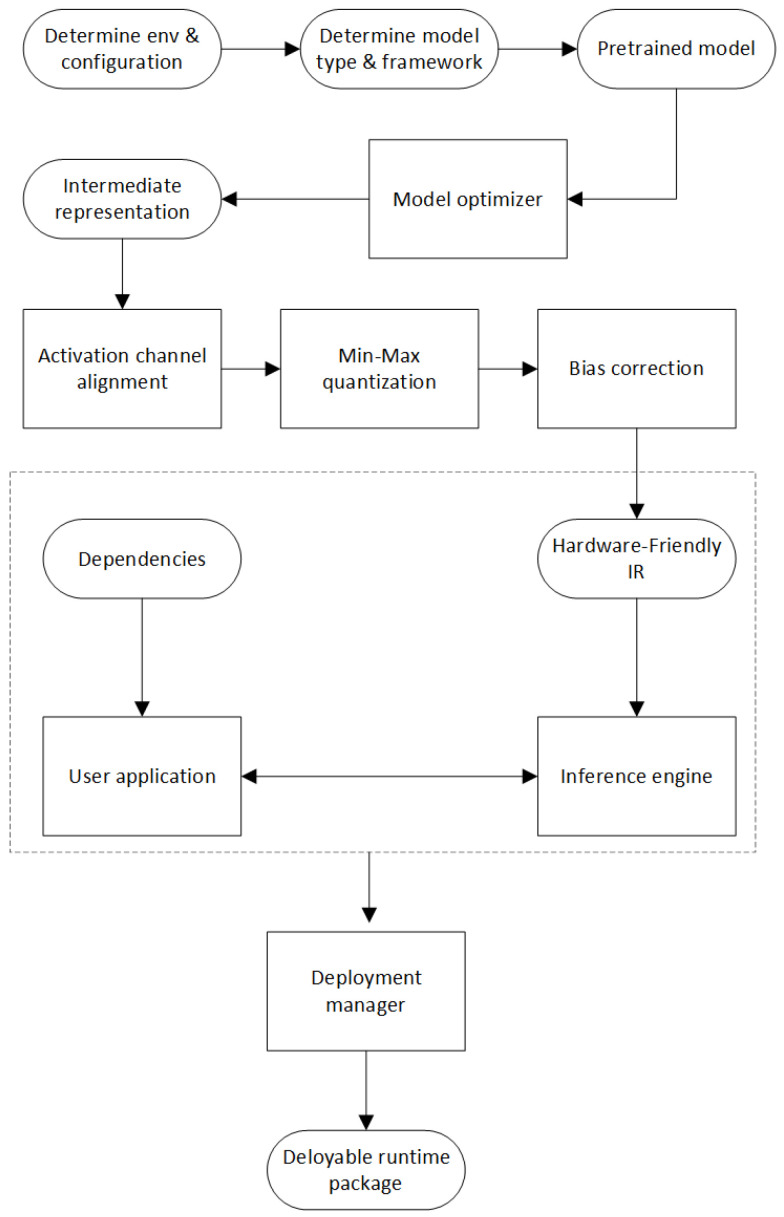
Detailed deployment process of the OpenVINO toolkit.

Our edge deployment was performed under the OpenVINO framework. OpenVINO is capable of deploying high-performance computer vision applications on edge gateways and contains a powerful toolkit for rapid development of computer vision applications that can run on edge gateways. The detailed deployment process of the OpenVINO toolkit is shown in Figure 12.

(1) Pretrained models: The OpenVINO toolkit comes with large number of pretrained networks that are specially trained and tuned for specific computer vision tasks. These models have been adopted in the intermediate representation (IR) format.

(2) Model optimizer: It converts models from multiple different frameworks into IR format and uses them with the inference engine to reduce the size and complexity of the model, and reduces memory and computing pressure.

(3) Inference engine: It is responsible for actual reasoning. The inference engine works on models created using the model optimizer or obtained from pretrained models. This tool provides hardware-based optimizations to further improve IR format models.

## 4. Result and Discussion

### 4.1. Dataset Description

The Chinese city parking dataset (CCPD) is a large-scale domestic parking lot license plate dataset created by a team from the University of Science and Technology of China for license plate detection and recognition. The CCPD2020 dataset mainly includes new energy license plate data in various lighting conditions, tilt angles, and weather conditions [42]. The images are stored in .jpg format and have a resolution of 1242 × 375. The cameras of the charging piles are used to record surveillance videos of vehicles entering and leaving the parking spaces of the charging piles at different time periods and under different weather conditions for fair comparison. The frame processing of these monitoring videos is carried out. To ensure the objectivity and authenticity of the performance evaluation, the real data of the vehicle were collected by manually retrieving the bicycle speed in the video clips, and the real-time monitoring dataset of the charging pile was constructed, including vehicles entering and exiting the charging pile parking space, including the new energy license plate dataset under different brightness, different tilt angles, and different weather conditions, to minimize potential data errors. The image resolution was 1032 × 1260, and it was saved in .jpg format. Table 1 introduces the numbers of training, testing, and validation sets for the two datasets.

### 4.2. Model Training

When training YOLOv7 on an Ubuntu 18.04 workstation, PyTorch 1.12 was used as the model framework. The OpenVINO range of EC devices supports CUDA 10.2 to speed up model training and model reasoning. The cost of obtaining labeled datasets for LPR was high, and there was a large amount of unlabeled data. In terms of feature distribution, both marked data and unmarked data had the same feature distribution. To make full use of unlabeled data labeling to improve the feature learning ability of the model, an OD framework for SSL was proposed in this paper. The Edge–LPR model was trained firstly using a modest quantity of marked data. After reaching stability, labels for unmarked data were generated by self-training.

The self-training algorithm required two sample sets, $Labeled=\left\{\left(x_{i},y_{i}\right)\right\}$, $Unlabeled=\left\{x_{j}\right\}$ and in quantity L<<U.

(1) A classification strategy *F* was generated with marked data.

(2) The classification strategy *F* was used to classify unmarked data and calculate the error.

(3) A subset of unmarked data was selected, and a label was added if the error was small.

(4) The above steps were repeated until the unmarked data were an empty set.

The marked data were continuously selected from the unmarked data to add samples with good performance. The algorithm calculation strategy of the subset was continuously updated. An optimal calculation strategy was obtained. In the self-training process, an entropy-based regularization loss term was added to the Edge–LPR network loss function to make the prediction result more accurate. The co-training training method was used to predict the unmarked data on the training model, and the pseudo-mark data were generated. Finally, we generated a more accurate test model. It can learn more feature information from the data, and the prediction results were more accurate. The specific parameters of model training are shown in Table 2.

Precision, recall rate, FPS, mAP, and $F_{1}$ score are the major assessment metrics for LPR. There are four categories for what is associated with license plate detection: TP represents positive samples that are correctly classified, FP represents positive samples that are misclassified, FN represents negative samples that are misclassified, and TN represents negative samples that are correctly classified.

The proportion of samples that are correctly classified as positive to all samples that are detected is referred to as precision. Recall is the proportion of such samples that are correctly categorized and recognized to the complete target test set of such samples. Additionally, the algorithm’s performance on the target frame is determined and evaluated using the missed detection rate. The F1 score considers both the precision and recall of the classification model.

(13) Re=TPTP+FN

(14) $$\mathrm{Pr}=\frac{TP}{TP+FP}$$

All possible parameters of precision and recall were tested by us to construct precision–recall curves. The area under the curve, which had a value range of 0 to 1, was used to calculate the network’s average precision:

(15) $$AP=\int_{0}^{1}p\left(r\right)dr$$

(16) F1=2·Pr·RePr+Re

Different compression thresholds were used to reduce the model size; the pruning percentages of 0.5, 0.8, and 0.9 were set, and the models were trained sparsely. Table 3 and Table 4 give the impact of the model at different pruning thresholds. When the compression threshold is 0, it is the original model without model compression.

**Figure 13:**
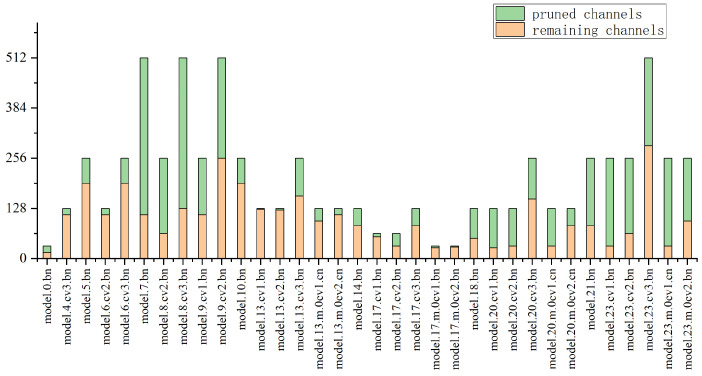
The channels pruned by the model BN layer and the number of reserved channels when the pruning rate is 0.5.

Figure 13 illustrates how much channel pruning occurs in various BN layers. When more channels are kept, this layer becomes even more crucial. Only a part of the most crucial channels are retained by those BN layers, and all other channels are pruned. These BN layers have little impact on the entire neural network.

To effectively demonstrate the superiority of the algorithm proposed in this paper for license plate detection, a comparative experiment was conducted on the same hardware device with the mainstream detection algorithms SSD, Faster RCNN, YOLOv4 series, YOLOv5 series, YOLOX series, YOLOv7 series, EfficientDet, deep feedforward network (DFF) and Edge–LPR system. The comparison results are shown in Table 5. We compared the state-of-the-art detection algorithms, and the results are shown in Table 6. The number of params and FLOPs of our Edge–LPR network was significantly less than that of other detectors, and the detection speed can reach 187.6 FPS.

**Figure 14:**
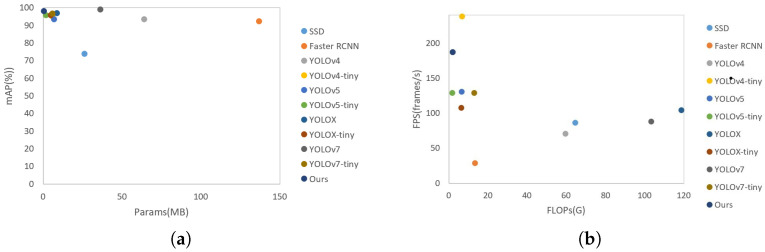
Illustration of the trade-off among mAP and the number of parameters, the number of FLOPs, and the inference speed. (**a**) mAP–params curve. (**b**) FPS–FLOPs curve.

This paper give two plots in Figure 14 that show the link between the number of mAPs in relation to parameters and the number of FLOPs in relation to inference speed to illustrate the trade-off between accuracy and effectiveness. The Edge–LPR successfully strikes a balance between accuracy, parameter quantity, FLOPs, and inference speed.

For edge model inference, power consumption is a key issue to ensure its effectiveness. OpenVINO was selected as the edge gateway platform for model inference in this paper. Model inference in lightweight networks was held using GPU and CPU, which significantly reduced the vulnerability of embedded devices to memory resources while also preserving great detection accuracy. The aforementioned network framework was retrained using the real-time monitoring dataset of the charging station, and accuracy was noticeably increased. The network model’s final scale and computational complexity had marginally decreased, while the speed of inference had marginally increased. The number of Edge–LPR parameters can be greatly decreased by compressing the trunk layer and reconstructing the neck. This can accomplish quick multiobjective detection of vehicle edge scenarios while also better meeting real-time needs.

**Figure 15:**
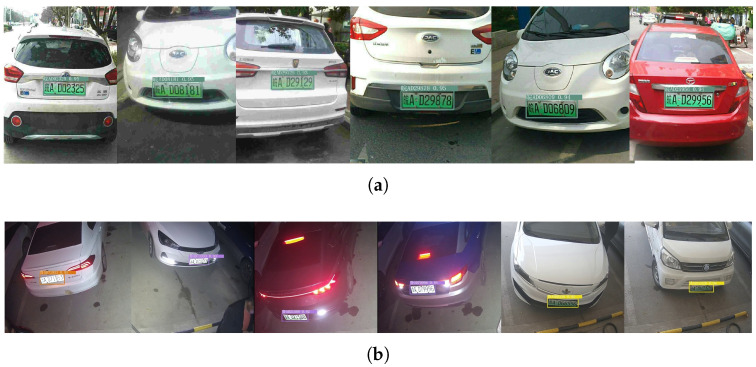
Compression model test result sample. (**a**) Detection results of CCPD2020 dataset. (**b**) Test results in charging pile environment.

**Figure 16:**
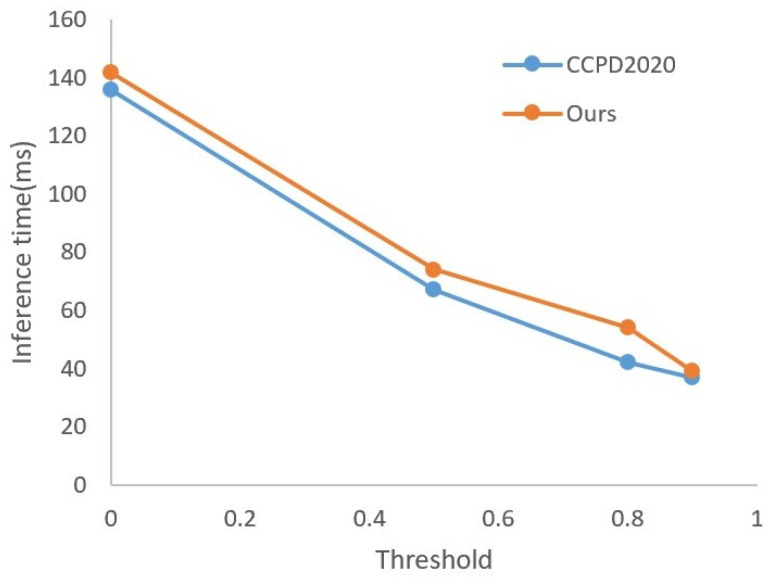
Inference time on different datasets before and after YOLOv7 pruning.

Edge–LPR can accurately detect license plate targets at different distances, different lights, and different environments in Figure 15, and multiple targets in one picture can be detected. Figure 16 shows the reasoning time of Edge–LPR under different compression thresholds in the CCPD2020 dataset and the charging station real-time monitoring dataset. The higher the threshold, the faster the inference time. When the compression threshold is 0.9, the fastest inference time of the model is around 40 ms.

The trained LPR model was reasoned by using the OpenVINO deployment on the Intel i7 9th generation processor, the Python deployment on Nvidia RTX2070, and the OpenVINO deployment on the gateway of EC. The CCPD license plate dataset was tested, and the comparative data of different hardware and inference frameworks are shown in Table 6.

The OpenVINO framework has a faster inference time than the PyTorch framework when running on identical hardware. The Intel i7 9th generation processor outperforms the Intel second-generation computational neural network when using the OpenVINO framework for inference, and the OpenVINO framework can produce real-time results when installed on gateways. All recognitions’ average accuracy (mAP) falls within a manageable error range.

On EC devices, the CUDA 10.1 was integrated into the Python 1.6 training framework to speed up model training and model reasoning. The 18.04 version of the Ubuntu system setting was used for our work. The GPU, memory, gigabit floating-point computations per second (GFLOPS), thermally designed power, and manufacturing stages for each system combination are thoroughly broken down in Table 7. The DL workstation in the cloud has a thorough hardware setup that comprises an Intel i7 9th generation CPU processor, AMD Ryzen 7 5800H, an NVIDIA RTX 2070 graphics card, and 128 GB of Intel second-generation computing stick (NCS2) memory. In terms of EC, the processing power and power consumption of EC hardware are, respectively, 1.7% and 7.8%.

To verify the advantages of low data transmission and low latency of edge computing, this paper conducts tests on the NCS2 platform and cloud computing platform, respectively, and records the amount of data transferred and the time spent in Table 8.

Cloud computing license plate detection usually requires five steps: (1) capture the image; (2) upload; (3) process in the cloud; (4) download the detection image and results; (5) output the detection results at the edge. The cloud host hardware configuration is NVIDIA RTX2070, and the network test environment is set up with a bandwidth of 200 M. The edge gateway has license plate detection capabilities; therefore, there is no need to download the results. After the image is captured and processed, the result is returned directly.

The process from capturing the license plate image to outputting the license plate information is defined as a response cycle. With cloud computing, it takes 23 ms to capture an image. The uploaded process takes 104 ms, which include packaging the detection image into JavaScript Object Notation (JSON) format data and network latency. The processing time is 28 ms, including API routing analysis, image detection, and result output. It takes 89 ms to download the detection images and results. This process includes network transmission and terminal data analysis. Finally, the edge device takes 34 ms to output the license plate information. Therefore, the response cycle of cloud computing is 278 ms. In contrast, if edge computing is used, network I/O time is significantly reduced, and the response cycle only includes capturing the image (21 ms), edge processing (96 ms), and outputting the result (32 ms). Therefore, the detection cycle only takes 149 ms. Edge computing solutions reduce latency by 46% compared to cloud computing. The focus of the above experiments is to identify the differences between the two computing modes, and the impact of network infrastructure instability is beyond the scope of this paper.

**Figure 17:**
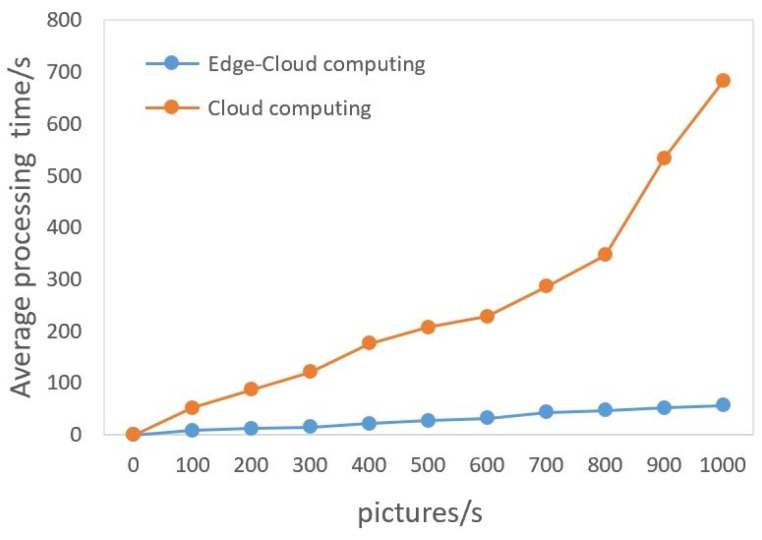
Comparison of the number and latency of uploaded images between cloud computing and edge cloud computing methods.

Figure 17 shows a comparison of the number and latency of uploaded images between cloud computing and EC computing methods. Edge–LPR was deployed on cloud computing workstations and had high inference ability. We tested for transmission latency, which had been shown to slow down edge processing speed, and is necessary for cloud computing systems.When the volume of photos grows, the transmission strain caused by cloud computing causes latency to accelerate, and it is a challenge to guarantee real-time and quick performance. This phenomenon shows that the EC cooperation architecture provides benefits for deep edge learning, and the timeliness of edge gateways has been fully utilized.

## 5. Conclusions

Edge–LPR is an EC and lightweight model LPR system based on SSL, which is proposed in this paper. Edge–LPR used the method of SSL to solve the problem of insufficient label data in actual work, greatly improved work efficiency, and supported the LPR algorithm based on DL in EC scenarios. To improve the accuracy of multiscale prediction, the pruned feature extraction network and the compressed feature fusion network were coupled together without reducing the accuracy of the model. In contrast to conventional LPR methods, it used cloud computing to upload data gathered at the network’s edge and continually update the overall network model. The real-time recognition capabilities of EC devices were improved. Edge–LPR maintained a better balance between the YOLO algorithm’s rapidity and precision on edge gateways, and it is suitable for tiny edge gateways. Additionally, we incorporated Edge–LPR into more platforms and situations for intelligent transportation systems and applied it to a greater variety of EC devices.

## Figures and Tables

**Table 1:** Dataset description.

Dataset	CCPD 2020	Ours
Classes	2	2
Train dataset	5769	2652
Test dataset	1001	650
Validation dataset	5006	1847

**Table 2:** Model parameter settings.

Parameter	Values
Weight decay	0.0005
Batch size	16
Learning rate	0.01
Epoch	200

**Table 3:** Effects of different thresholds on the compression of the YOLOv7s model in the CCPD2020 dataset.

Threshold	0	0.5	0.8	0.9
Params (MB)	36.6	19.5	12.2	8.1
Model storage size (MB)	74.8	37.2	14.7	8.2
Speed/GPU (ms)	17	12	11	9
Speed/CPU (ms)	154	86	58	41
mAP (%)	97.1	96.5	96.2	95.6

**Table 4:** Effect of different thresholds on the compression of YOLOv7s model in real-time monitoring dataset of charging pile.

Threshold	0	0.5	0.8	0.9
Params (MB)	36.6	23.5	12.3	8.3
Model storage size (MB)	74.8	37.3	14.0	8.1
Speed/GPU (ms)	26	18	15	11
Speed/CPU (ms)	168	98	64	52
mAP (%)	96.1	95.5	95.2	94.6

**Table 5:** Comparison with the state-of-the-art detection algorithms.

Detection Algorithm	mAP (%)	F1	Params (MB)	FLOPs (G)	Speed (FPS)
SSD	73.82	0.725	26.285	64.818	86.174
Faster RCNN	92.25	0.891	137.057	13.52	28.405
YOLOv4	93.48	0.872	64.106	59.851	70.622
YOLOv4-tiny	95.07	0.916	5.924	6.862	238.151
YOLOv5	93.48	0.928	7.093	6.76	130.069
YOLOv5-tiny	95.77	0.919	1.821	1.796	128.555
YOLOX	96.92	0.928	8.968	118.965	104.149
YOLOX-tiny	95.65	0.931	5.056	6.41	107.208
YOLOv7	98.83	0.945	36.49	103.5	87.624
EfficientDet	95.62	0.938	3.9	2.5	16.25
DFF	87.68	0.897	144	160	35.48
Edge–LPR (ours)	95.6	0.914	1.1	5.3	187.6

**Table 6:** Comparison with the other algorithms.

Detection Algorithm	mAP (%)	Speed (FPS)
CA-CenterNet [17]	96.8	52.7
LSV-LP [18]	89.3	112.56
P2OD [25]	97.52	108
Li et al. [27]	95.59	132.76
MFLPR-Net [30]	92.02	54
Edge–LPR (Ours)	95.6	187.6

**Table 7:** Comparison of different hardware performance.

Computer Platform	Memory	GLOPS (FP16)	Thermal Design Power	Manufacturing Process
AMD Ryzen 7 5800H	8 GB 64 bit DDR4	4600	45 W	7 nm
Intel Core i711700K	8 GB 64 bit LPDDR4x	450	125 W	10 nm
NVIDIA RTX2070	11 GB 35GDDR5X	7500	175 W	12 nm
NCS2	128 G	4	1.5 W	28 nm

**Table 8:** Edge and cloud computing response times.

Step	Edge Computing	Cloud Computing
Step 1	Capturing images 21 ms	Capturing images 23 ms
Step 2	Edge computing 96 ms	Uploading original images 104 ms
Step 3	Output results 32 ms	Cloud computing 28 ms
Step 4	–	Result returned 89 ms
Step 5	–	Output results 34 ms
Response cycle	148 ms	278 ms

## Data Availability

Part of the data based on this research institute was accessed from the CCPD2020 public database and can be downloaded through the following link: https://github.com/detectRecog/CCPD, accessed on 15 October 2022.
